# The Autophagy Regulator p62 Controls PTEN-Dependent Ciliogenesis

**DOI:** 10.3389/fcell.2020.00465

**Published:** 2020-06-10

**Authors:** Hyowon Mun, Eun Ji Lee, Minah Park, Goo Taeg Oh, Jong Hoon Park

**Affiliations:** ^1^Department of Biological Science, Sookmyung Women’s University, Seoul, South Korea; ^2^Department of Biology, Ewha Women’s University, Seoul, South Korea

**Keywords:** autophagy, cilia, ciliogenesis, SQSTM1/p62, PTEN

## Abstract

Autophagy is a catabolic process required for maintaining intracellular energy homeostasis. It eliminates harmful proteins and recycles functional macromolecules back into the cell via cargo breakdown. Autophagy is generally suppressed under fed conditions and induced by serum starvation; therefore, it is considered to be a nutrient-sensing mechanism. Cilia, finger-like organelles harboring multiple receptors along their surface, are energy-sensing structures that are also triggered by serum deprivation. Herein, we verified the effect of autophagy alterations on cilia assembly and the specific underlying mechanisms. Autophagy flux altered either by drugs or autophagy-targeting siRNAs strongly inhibited ciliogenesis, and this inhibition was affected by p62, an autophagy regulator, via Pten/Dvl2/AurKA signaling.

## Introduction

Autophagy is an intracellular process that degrades non-functional/damaged proteins under serum starvation or stress conditions and maintains cellular homeostasis by eliminating harmful proteins and recycling functional breakdown products. There are three types of autophagy: macroautophagy, microautophagy, and chaperon-mediated autophagy (Kaur and Debnath, 2015). Macroautophagy has been well described and uses double membraned-structures known as autophagosomes to sequester cargo proteins. The process begins with the formation of a phagophore which expands via the recruitment of multiple autophagy-related (ATGs) proteins. The ULK1-Atg13-FIP200 complex is involved in the initial stage of the process and directly stimulates the Beclin 1 phosphorylation (Russell et al., 2013). Class III phosphatidylinositol 3-kinase (PI3K) and Beclin 1 are required for vesicle nucleation, followed by two different conjugation systems mediated by ubiquitin-like proteins. The first is the Atg12-Atg5-Atg16L complex: Atg12, an ubiquitin-like protein, is irreversibly bound to Atg5 by the E1-like enzyme Atg7 and forms a final complex with Atg16L. It is directed to the outer surface of the phagophore membrane and dissociates immediately after the formation of the enclosed, cup-shaped autophagosome (Panda et al., 2015). LC3 (microtubule-associated protein light chain 3) is an ubiquitin-like protein essential for autophagosome maturation. Pro-LC3 primarily undergoes C-terminal cleavage by the cysteine protease Atg4 and conjugation with phosphatidyl-ethanolamine (PE), resulting in the lipidated form of LC3 (LC3-II; Ichimura et al., 2000; Fullgrabe et al., 2014). LC3-II is localized on the autophagosomal membrane and assists with lysosomal fusion (Nakatogawa et al., 2007). The mature autophagosome encounters and fuses with the lysosome, thereby delivering cargo that is broken down by acidic lysosomal enzymes.

Cilia are antenna-like organelles that extend from the surface of eukaryotic cells. They are ubiquitous in almost every vertebrate cell and are highly conserved between species. Cilia have microtubule-based structures consisting of peripheral microtubule doublets with or without a central pair of microtubules (9 + 2 motile cilia or 9 + 0 non-motile cilia, respectively). Motile cilia promote movement (such as clearing airways in the lungs), whilst non-motile cilia (hereafter referred to as primary cilia) function as cellular sensory antennae in response to external stimuli (Brown and Witman, 2014). Specific receptors, including Hedgehog and PTCH, are clustered within cilia; therefore, functional cilia defects can lead to human diseases known as ciliopathies. Cilia formation is directly associated with the cell cycle. The process begins with axoneme nucleation from a centriole-derived basal body during interphase, followed by cilia assembly and extension. Upon cell cycle exit, the centrosome migrates to the cell surface and distal appendages on the docked mother centriole fuse with the plasma membrane (Kobayashi and Dynlacht, 2011; Basten and Giles, 2013; Izawa et al., 2015). The ciliary axoneme is then extended and cilia are surrounded by a ciliary membrane (Avasthi and Marshall, 2012). Multiple proteins including motor proteins, intraflagellar transport (IFT) particles, and their cargos mediate these processes (Hao and Scholey, 2009). Intraflagellar transport proteins move bi-directionally along ciliary axonemes (from the base to tip, or vice versa) carrying proteins necessary for cilia assembly. Cilia are resorbed when core IFT particles and motors are carried back to the basal body prior to mitotic entry (Rosenbaum and Witman, 2002; Scholey, 2008; Plotnikova et al., 2009).

Both autophagy and ciliogenesis are concurrently triggered by serum deprivation; thus, are essential nutrient-sensing mechanisms for managing intracellular energy defects. Recently, potential molecular links have been reported between autophagy and primary cilia; however, the first two studies have revealed the different results showing dual role for autophagy in ciliogenesis control. One group observed that autophagy induced ciliogenesis by degrading OFD1 (oral-facial-digital syndrome 1) from centriolar satellites (Tang et al., 2013). Another proposed that basal autophagy inhibits ciliogenesis by removing ciliary proteins, such as Ift20, which are involved in elongation (Pampliega et al., 2013). These controversial studies prompted us to investigate the precise correlation between autophagy and ciliogenesis.

Herein, we tried to identify signaling modules linking ciliogenesis and autophagy, which are simultaneously stimulated by nutrient stress, based on current studies. One of them has identified the role of phosphatase and tensin homolog (PTEN) to stabilize cilia through dephosphorylation of Dishevelled2 (Dvl2) which is finally led to Aurora kinase A (AurKA)-mediated cilia disassembly (Gao and Chen, 2010; Shnitsar et al., 2015). In addition, several studies have also revealed a potential role of PTEN in autophagy regulation (Arico et al., 2001; Niu et al., 2016; Rosenfeldt et al., 2017). Therefore, we attempted to verify whether PTEN-DVL2-AurKA signaling could regulate cilia by altering autophagy. Pten expression increased in a time-dependent manner during serum starvation; however, its genetic silencing strongly inhibited both ciliogenesis and autophagy, as indicated by changes in p62 accumulation. p62 is an autophagy cargo protein with multiple domains that interact with LC3 and ubiquitin; thus, p62 also delivers other ubiquitinated cargos to autophagosomes (Liu et al., 2016). p62 labeling is widely used to track autophagy since its expression is reduced by successful autophagy. High p62 accumulation and enhanced Dvl2/AurKA levels were observed in Pten-silenced cells under both normal and fasting conditions. These effects were recovered by p62 knock-down, suggesting that the autophagy factor p62 links the cilia-inhibiting effect of Pten silencing with Dvl2-AurKA signaling.

## Materials and Methods

### Cell Culture

NIH/3T3 mouse embryonic fibroblasts were obtained from the Korean Cell Line Bank and cultured in DMEM (LM001-05, Welgene) supplemented with 10% FBS (fetal bovine serum; 26140-079, Gibco) and 1% penicillin–streptomycin (LS 202-02, Welgene). Under normal conditions, NIH/3T3 cells were cultured in 10% FBS, whilst under serum starvation conditions they were cultured in 0.5% FBS for 24 h. All cells were incubated in a humidified incubator with 5% CO_2_ at 37°C. To regulate autophagy *in vitro*, cells were treated with 5 nM of rapamycin (R8781, Sigma-Aldrich) and 50 uM of chloroquine (CQ; C6628, Sigma-Aldrich) for 4 h. Drugs were treated after 24 h serum starvation when it is necessary to induce ciliogenesis.

### Western Blot

Proteins were isolated from cells and kidney tissue using NucleoSpin RNA/Protein kits (Macherey-Nagel). Protein concentration was calculated using the Bradford assay with bicinchoninic acid (B9643, Sigma) and copper sulfate (C2284, Sigma) solutions. Proteins were separated by 8–15% sodium dodecyl sulfate polyacrylamide gel electrophoresis (SDS-PAGE) and transferred to polyvinylidene fluoride (PVDF) membranes (AE-6667-P, ATTO). The following primary antibodies were used in this study: Atg5 (#12294, Cell Signaling Technology), LC3 A/B (#12741, Cell Signaling Technology), p62 (#5114 and #23214, Cell Signaling Technology), Pten (#9552, Cell Signaling Technology), pDVL (ab124933, Abcam), Dvl2 (#3224, Cell Signaling Technology), and β-actin (A300-491A, Bethyl Laboratories).

Membranes were blocked with 5% skimmed milk in PBST (1 × PBS with 1% Tween-20), incubated with primary antibodies diluted in 1% skimmed milk with PBST overnight at 4°C, washed with PBST, and incubated with secondary antibodies in 2% skimmed milk for 1 h at room temperature. Protein bands were detected using enhanced chemiluminescent reagents (WSE-7120 EzWestLumi plus, ATTO) and band intensity was visualized using an LAS-3000 instrument (Fujifilm).

### Immunofluorescence Microscopy

Cells incubated on coverslips were fixed in 4% paraformaldehyde in PBS or cold methanol for 10 min, blocked and permeabilized for 15 min, and incubated with the following primary antibodies at 4°C overnight: anti-acetylated α tubulin (T6793, Sigma-Aldrich and #5335, Cell Signaling Technology), anti-γ tubulin (T6557, Sigma-Aldrich) and p62 (#5114 and #23214, Cell Signaling Technology). The following day, cells were stained with DAPI for 15 min and incubated with FITC-conjugated rabbit anti-mouse IgG (sc-358916, Santa Cruz Biotechnology), goat anti-mouse IgG Alexa 488 (A11029, Thermo Fisher Scientific), or goat anti-rabbit IgG Alexa 594 (A11037, Thermo Fisher Scientific) antibodies for 2 h at room temperature. Finally, the slides were mounted with mounting solution (S3023, Dako) and visualized by confocal microscopy (LSM-700, Carl Zeiss). For histological analysis, sections were counter-stained with hematoxylin & eosin (H&E) and renal collecting duct was observed by staining with rhodamine-conjugated DBA (Vector Laboratories). p62 and cilia were quantified by the puncta-to-nucleus ratio, and finally analyzed using macro program of Image J which allows measure the average number and size of GFP puncta per cell.

### siRNA Transfection

Cells were transfected with control small interfering RNA (siRNA; 5′-AUG AAC GUG AAU UGC UCA ATT-3′/5′-UUG AGC AAU UCA CGU UCA UTT-3′, ST pharm and sc-37007, Santa Cruz Biotechnology) or siRNA targeting Atg5 (sc-41446), PTEN (sc-36326), and SQSTM1 (sc-29828) using Liopfectamine RNAiMAX Reagent (Invitrogen) according to the manufacturer’s instructions.

### Quantitative Real-Time PCR

Messenger RNA (mRNA) was isolated using a NucleoSpin^®^ RNA/Protein kit (Macherey-Nagel) and reverse-transcribed (2 μg) using a mixture of M-MLV Reverse Transcriptase (M170B, Promega), RNasin^®^ Ribonuclease Inhibitor (N211A, Promega), 100 nM oligo-dT, and 2.5 mM dNTP. Quantitative real-time PCR (qRT-PCR) was conducted using a qPCRBIO SyGreen Mix Lo-Rox (PB20, PCR Biosystems) according to the manufacturer’s instructions with the following primers: mouse *Pten* (forward: 5′-GAA AGG GAC GGA CTG GTG TA-3′, reverse: 5′-ACT CCC TTT TTG TCT CTG GT-3′), and mouse β-actin (forward: 5′-GAC GAT GCT CCC CGG GCT GTA TTC-3′, reverse: 5′-TCT CTT GCT CTG GGC CTC GTC ACC-3′).

### Statistical Analysis

All *in vitro* data were obtained from a minimum of three independent experiments, and more specifically, all immunoblot data were quantified with three to five gels. It was analyzed by two-tailed *t*-tests in GraphPad InStat (Graphpad software), and reported as the mean ± Standard Deviation (SD). *P* < 0.05 was considered statistically significant (^∗^*P* < 0.05, ^∗∗^*P* < 0.01, and ^∗∗∗^*P* < 0.001).

## Results

### Inhibiting Autophagy Reduces Ciliogenesis

**FIGURE 1:**
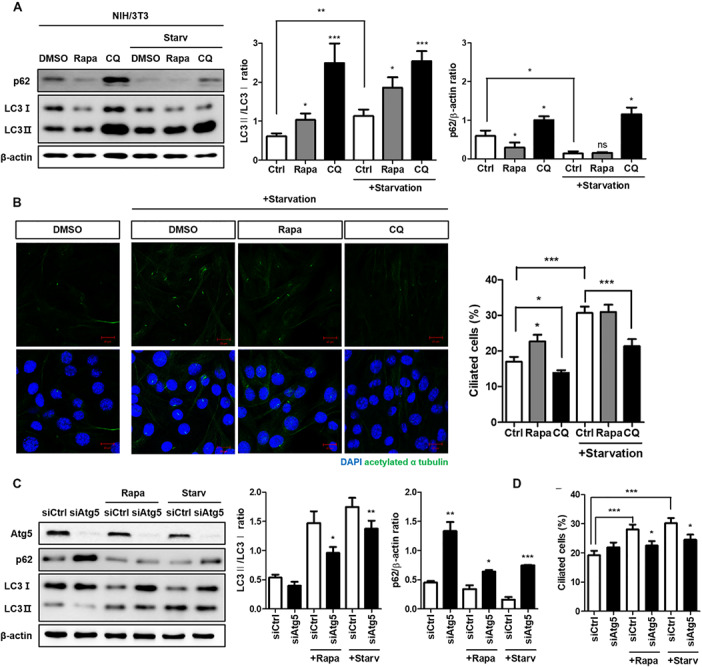
Reduced cilia formation by autophagy inhibition. **(A)** Effects of autophagy drugs (5 nM rapamycin, 50 μM chloroquine), which were treated after 24 h serum starvation, on conversion of LC3-I into LC3-II. **(B)** Changes in the number of ciliated cells under autophagy regulation. The percentage of ciliated cells, which was successfully induced by serum starvation (0.5% FBS, 24 h), were reduced by autophagy inhibitor (CQ, 4 h) treatment. **(C)** Dysregulated autophagy flux in *Atg5*-silenced NIH/3T3 cells. It was monitored by the inhibited LC3 conversion under autophagy induction (either by rapamycin treatment or serum starvation) compared to siCtrl-transfected cells. **(D)** Effect of *Atg5* silencing with autophagy stimulation on ciliogenesis. The number of ciliated cells which was quantified by cilia-to-nucleus ratio was significantly reduced in *Atg5*-silenced cells. All data were obtained from a minimum of three independent experiments. Statistics analyzed by two-tailed *t*-tests, and *P* < 0.05 was considered statistically significant (*P < 0.05, **P < 0.01, ***P < 0.001).

Both autophagy and ciliogenesis are considered as nutrient-sensing mechanisms which is concurrently stimulated by nutrient stress; therefore, we examined the condition which simultaneously induced autophagy and ciliogenesis. Cells treated with 0.5% FBS for 24 h increased autophagy flux as well as the number of ciliated cells (Supplementary Figure S1). To identify the potential molecular link between them, we examined whether the autophagy-targeting drugs rapamycin and CQ could affect ciliogenesis. Rapamycin is a well-known autophagy inducer, whilst CQ inhibits autophagy by preventing autophagosome-lysosome fusion. It accumulates autophagosome, therefore, autophagosomal membrane protein LC3 is increased by CQ (Galluzzi et al., 2017; Mauthe et al., 2018). The ratio of LC3-II/LC3-I expression was increased by treatment with 5 nM rapamycin for 4 h and was further enhanced by serum starvation. In addition, high LC-II accumulation was observed in cells treated with 50 μM CQ for 4 h, indicating that drug treatment successfully inhibited autophagy (Figure 1A). Changes in the number of ciliated cells were observed in each group, with a large decrease in the CQ treated group compared to the DMSO-treated group (Figure 1B). To confirm how ATG gene alterations modulated ciliogenesis, we knocked-down the *Atg5* gene under either rapamycin treatment or serum starvation (0.5% FBS for 24 h). *Atg5* silencing reduced the conversion of LC3 into LC3-II, particularly under induced autophagy, reducing the number of ciliated cells (Figures 1C,D). Taken together, these results suggest that cilia assembly is modulated by alterations in autophagy.

### PTEN Is Accumulated During Serum Deprivation and Modulates Autophagy

**FIGURE 2:**
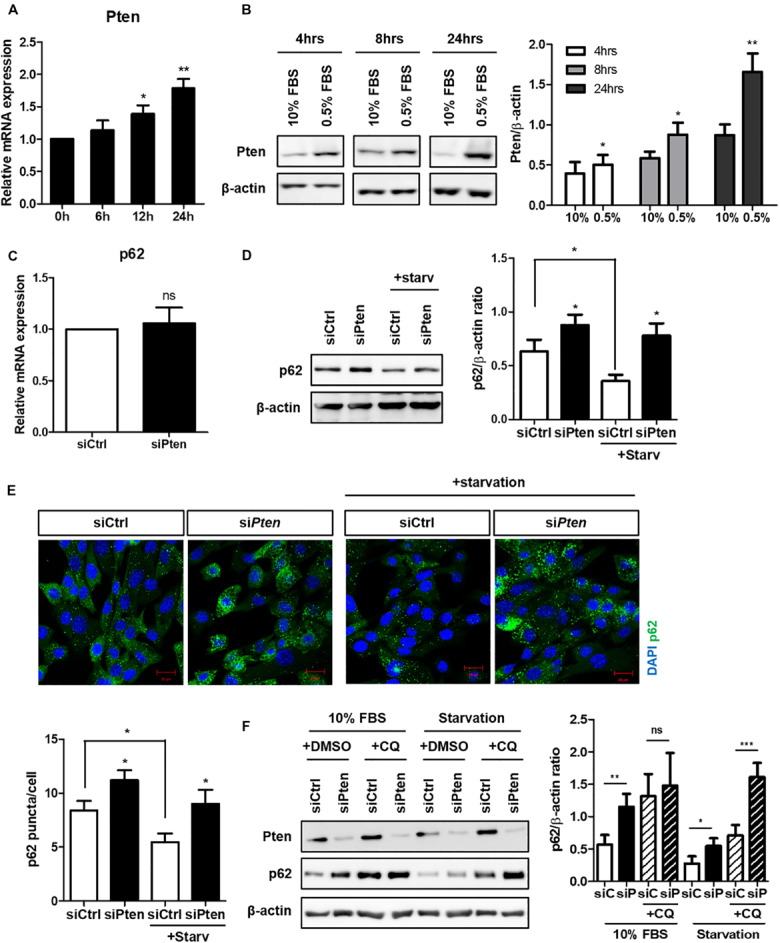
Increased Pten during serum starvation and p62 accumulation in Pten-silenced cell. **(A,B)** Increased Pten expression both in mRNA and protein level during serum starvation. **(C,D)** Changes of p62 at mRNA or protein level in Pten-silenced cells. **(E)** Observation of fluorescently-labeled p62 in *Pten* knock-down cells. p62 was highly accumulated in *Pten*-silenced cells compared to siCtrl-transfected cells under both basal (10% FBS) and serum starved condition (0.5% FBS, 24 h). **(F)** Changes of p62 flux in Pten-silenced cells with or without CQ treatment (50 μM, 4 h) under autophagy modulation. All data were obtained from a minimum of three independent experiments. Statistics analyzed by two-tailed *t*-tests, and *P* < 0.05 was considered statistically significant (*P < 0.05, **P < 0.01, ***P < 0.001).

Next, we attempted to identify the specific signaling modules via which autophagy regulates ciliogenesis. *Pten* was a candidate gene based on a previous study which demonstrated the critical role of the PTEN-DVL2 axis in the dynamic control of cilia (Shnitsar et al., 2015). *Pten* expression gradually increased during serum starvation and peaked at 24 h (Figures 2A,B). *Pten* did not affect *p62* at a transcription level, but increased p62 protein level (Figures 2C,D). To verify whether increased p62 in *Pten*-silenced cell was via autophagy, changes of fluorescent-labeled p62 puncta was monitored. p62 is an autophagy substrate, thus its clearance is considered a marker of successful autophagy induction. It was highly enhanced in *Pten*-silenced cell with or without serum starvation, indicating autophagy was inhibited by *Pten*-silencing (Figure 2E). To confirm autophagy-dependent p62 accumulation upon *Pten* knock-down, p62 flux was monitored by CQ treatment under autophagy modulation (Figure 2F). Chloroquine prevents lysosome acidification, resulting into the blockage of p62 degradation and allowing quantitation of the autophagy flux. Therefore, the higher increase after CQ treatment represents that the higher amount of p62 has been degraded by autophagy during the period of treatment. As results, the changes of p62 level was reduced by CQ in *Pten* knock-down cells (difference between 2nd and 4th bar) compared to siCtrl-transfected one (difference between 1st and 3rd bar), suggesting that *Pten*-silencing accumulated p62 mainly by the impaired autophagy control. On the contrary, accumulated p62 level was highly enhanced in *Pten*-silenced cells after CQ treatment (difference between 6th and 8th bar) under serum starvation. It indicated that other pathways including stress responses, which are autophagy-independently stimulated and accumulate p62 under nutrient stresses, also have been involved and affected intracellualr p62 levels.

### PTEN-Silencing Inhibits Ciliogenesis

**FIGURE 3:**
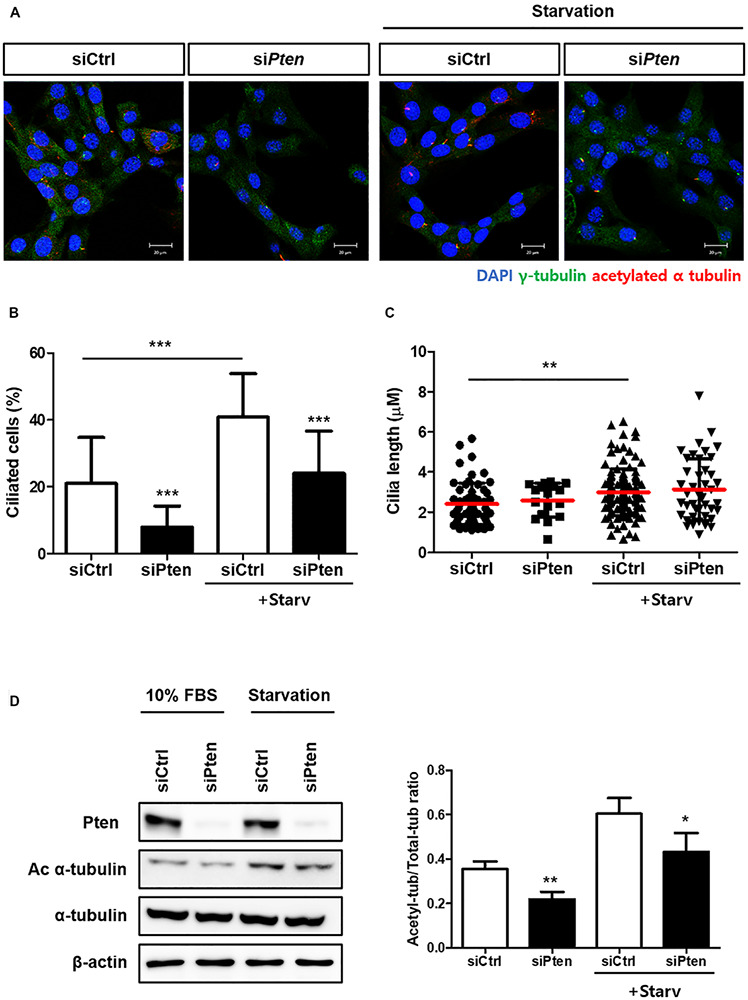
Inhibited ciliogenesis by Pten-silencing. **(A–C)** Changes in the percentage of ciliated cells and cilia length by *Pten* knock-down. *Pten*-silenced cells failed to induce cilia under serum starvation (0.5% FBS, 24 h). **(D)** Reduced acetylated alpha tubulin in *Pten*-silenced cells. Quantification was done by comparing the ratio of acetylated tubulin to total form. All data were obtained from a minimum of three independent experiments. Statistics analyzed by two-tailed *t*-tests, and *P* < 0.05 was considered statistically significant (*P < 0.05, **P < 0.01, ***P < 0.001).

To identify whether PTEN-silencing followed by the inhibited autophagy affects ciliogenesis, the changes of ciliated cells were observed with or without PTEN-silencing. As results, the percentage of ciliated cells with acetylated alpha tubulin was reduced when *Pten* was silenced; however, cilia length was not affected (Figures 3A–C). Moreover, the acetylated alpha-tubulin was successfully enhanced by serum starvation, while si*Pten*-transfected cells did not (Figure 3D), suggesting *Pten* silencing led to ciliary defects.

### SQSTM1/p62 Controls PTEN/DVL2/AurKA-Mediated Ciliogenesis

**FIGURE 4:**
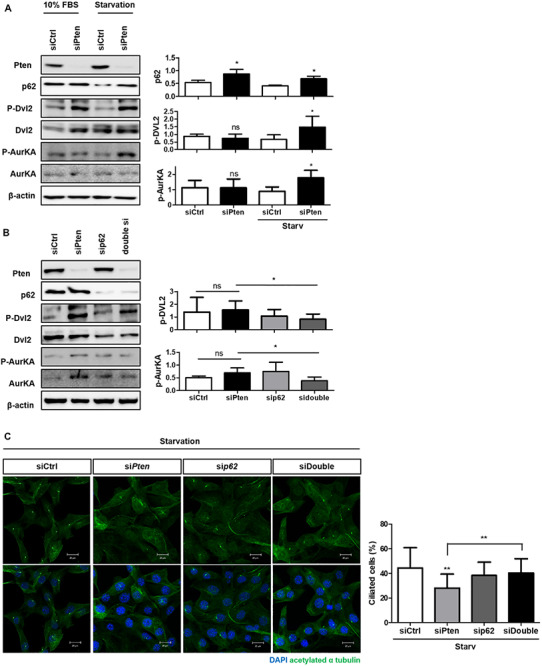
The role of SQSTM1/p62 in the PTEN/DVL2/AurKA-mediated ciliogenesis. **(A)** Monitoring p62 expression and Pten/Dvl2/AurKA activities under serum starvation (0.5% FBS, 24 h) via immunoblotting. Dvl2 and AurKA phosphorylation were highly enhanced with p62 accumulation in starved condition. **(B,C)** Recovery effect of p62-silencing on the percentage of ciliated cells and Dvl2/AurKA expression. Enhanced Dvl2/AurKA activities were restored by p62-silencing, and it finally led to increased number of ciliated cells under serum starvation (0.5% FBS, 24 h). All data were obtained from a minimum of three independent experiments. Statistics analyzed by two-tailed *t*-tests, and *P* < 0.05 was considered statistically significant (*P < 0.05, **P < 0.01, ***P < 0.001).

Finally, we verified whether p62 accumulation could directly affect cilia stabilization controlled by Pten through Dvl2-AurKA to identify the signaling modules linking cilia regulation and autophagy. *Pten* knock-down enhanced activities both of Dvl2 and AurKA with p62 accumulation in both normal and starved media (Figure 4A). We also tested whether inhibition of *p62* gene expression could recover the cilia-inhibiting effect of Pten/Dvl2/AurKA. Silencing p62 in *Pten* knock-down cells restored the percentage of ciliated cells and Dvl2/AurKA expression (Figures 4B,C).

These results indicate that p62 may regulate cilia via the Pten-Dvl2-AurKA signaling pathway as an upstream regulator of Dvl2.

## Discussion

Autophagy and ciliogenesis are two essential processes that manage nutrient stress by providing functional macromolecular components and signal transduction for energy production. Both are induced during serum starvation and maintain the intracellular energy balance. Functional correlation between the two processes has recently been reported; however, the first two reports were controversial. One suggested a positive relationship between autophagy and cilia whilst the other observed a negative correlation, suggesting that autophagy has an inhibitory effect on cilia formation. These studies have increased scientific interest concerning the correlation between autophagy and ciliogenesis in recent years. Early approaches examined whether the two processes affected each other under same stimulus, with research now focusing on identifying specific mediators. Recent studies have observed multiple Atg proteins near primary cilia and have shown that ciliary components are controlled by autophagy (Boehlke et al., 2010; Kim et al., 2015; Shin et al., 2015a, b; Wang et al., 2015; Orhon et al., 2016; Takacs and Proikas-Cezanne, 2016; Hsiao et al., 2018; Liu et al., 2018; Xu et al., 2018). Another candidate to modulate the interconnection between autophagy and ciliogenesis was AMP-activated protein kinase (AMPK). It was localized in basal body of cilia (Boehlke et al., 2010), and turned out to positively regulated autophagy via LKB1-AMPK-mTOR pathways (Kim et al., 2011; Mihaylova and Shaw, 2011; Roach, 2011). In addition, impaired autophagy observed in ciliopathies and vice versa have been increasingly reported. One of them was polycystic kidney disease (PKD). With hyper-activation of mammalian target of rapamycin (mTOR), which is a key negative regulator of autophagy, autophagy is commonly considered to be inhibited in PKD (Ravichandran and Edelstein, 2014). Besides, drugs targeting autophagy combined with rapamycin, has been recently shown a therapeutic effect, suggesting a new therapeutic target for the disease (Zhu et al., 2017). Multiple cancers, including breast cancer and renal cell carcinoma, are the other disease models which have been studied concerning the interplay between ciliogenesis and autophagy (Basten et al., 2013; Menzl et al., 2014; Ko et al., 2019). Cilia is known to be hardly detected in many cancer cells, whereas the role of autophagy is considered as the double-edged sword in cancer progression until now (Amaravadi et al., 2019; Romero et al., 2019; Yang et al., 2019). Studies regarding interplay between them is increasingly reported, however, its precise regulatory mechanisms are not yet clarified in cancer field. In these regards, although attempts to identify a functional relationship between autophagy and ciliogenesis are currently underway, the results remain contradictory and the precise underlying mechanisms have not been fully elucidated.

In this study, we attempted to verify whether cilia assembly was affected by altered autophagy and elucidate the specific underlying regulatory mechanisms. Using drugs or autophagy-targeting siRNAs, we found that cilia formation was positively influenced by autophagy, suggesting a reciprocal correlation between autophagy and ciliogenesis (Figure 1). We also attempted to identify potential signaling modules regulating cilia based on previous studies which suggested the Pten-Dvl2-AurKA axis. *Pten* silencing reduced the number of cilia, but not their length, and increased the accumulation of autophagy cargo p62 (Figures 2, 3). Moreover, enhanced Dvl2 and AurKA expression induced by *Pten* knockdown was successfully recovered by also silencing p62 (Figure 4). Altogether, we suggest that ATG protein p62 may regulate the mechanisms of Pten/Dvl2/AurKA-mediated ciliogenesis.

Our findings provide a potential novel mechanism by which autophagy regulates ciliogenesis and help understand the interplay between them. The specific molecular mechanisms via which p62 regulates Dvl2 in terms of autophagy flux should be elucidated. Besides, the increase of intracellular p62 levels by *Pten*-silencing what we have observed in the present study might be also affected by the stress responses stimulated under nutrient stress. p62 is a functional scaffold protein which is primarily involved in autophagy regulation in multi-level process by interacting with LC3 or ubiquitinated cargos via its own motifs. In addition to its roles in autophagy, p62 is an immediate-early response gene, which is activated by the multiple cellular stresses including nutrient stress, oxidative stress and immune responses, therefore, serves as a key signaling hub to maintain cell homeostasis (Katsuragi et al., 2015; Sanchez-Martin and Komatsu, 2018). Further investigations are needed to identify how autophagy-independent stress responses have involved in p62 accumulation by *Pten*-silencing under nutrient stress. Finally, identifying the functional significance of autophagy in ciliopathies (cystic diseases, polydactyly, skeletal abnormalities, etc.), and vice versa, might improve our understanding of this field.

## Data Availability Statement

The raw data supporting the conclusions of this article will be made available by the authors, without undue reservation, to any qualified researcher.

## Author Contributions

JP designed the study. HM, EL, and MP carried out the experiments and wrote the manuscript. GO discussed *in vivo* models with autophagy deficiencies. All authors approved the final version of the manuscript.

## Conflict of Interest

The authors declare that the research was conducted in the absence of any commercial or financial relationships that could be construed as a potential conflict of interest.

## Supplementary Material

FIGURE S1 Simultaneous induction of autophagy and ciliogenesis under serum starvation. **(A)** Increased number of ciliated cells by serum starvation induced by reducing FBS to 0.5% for 24 h. The number of ciliated cells were quantified by cilia-to-nucleus ratio. **(B)** Changes of autophagy flux through monitoring LC3 conversion into lipidated form (LC3-II) and the level of cargo protein p62 in starved condition (0.5% FBS, 24 h). All data were obtained from a minimum of three independent experiments. Statistics analyzed by two-tailed *t*-tests, and *P* < 0.05 was considered statistically significant (^∗^*P* < 0.05, ^∗∗^*P* < 0.01, ^∗∗∗^*P* < 0.001).
