# Supplementary figures and images for: The Autophagy Regulator p62 Controls PTEN-Dependent Ciliogenesis
Source: Front Cell Dev Biol. 2020 Jun 10;8:465. doi: 10.3389/fcell.2020.00465 (PMC7298063; doi:10.3389/fcell.2020.00465)

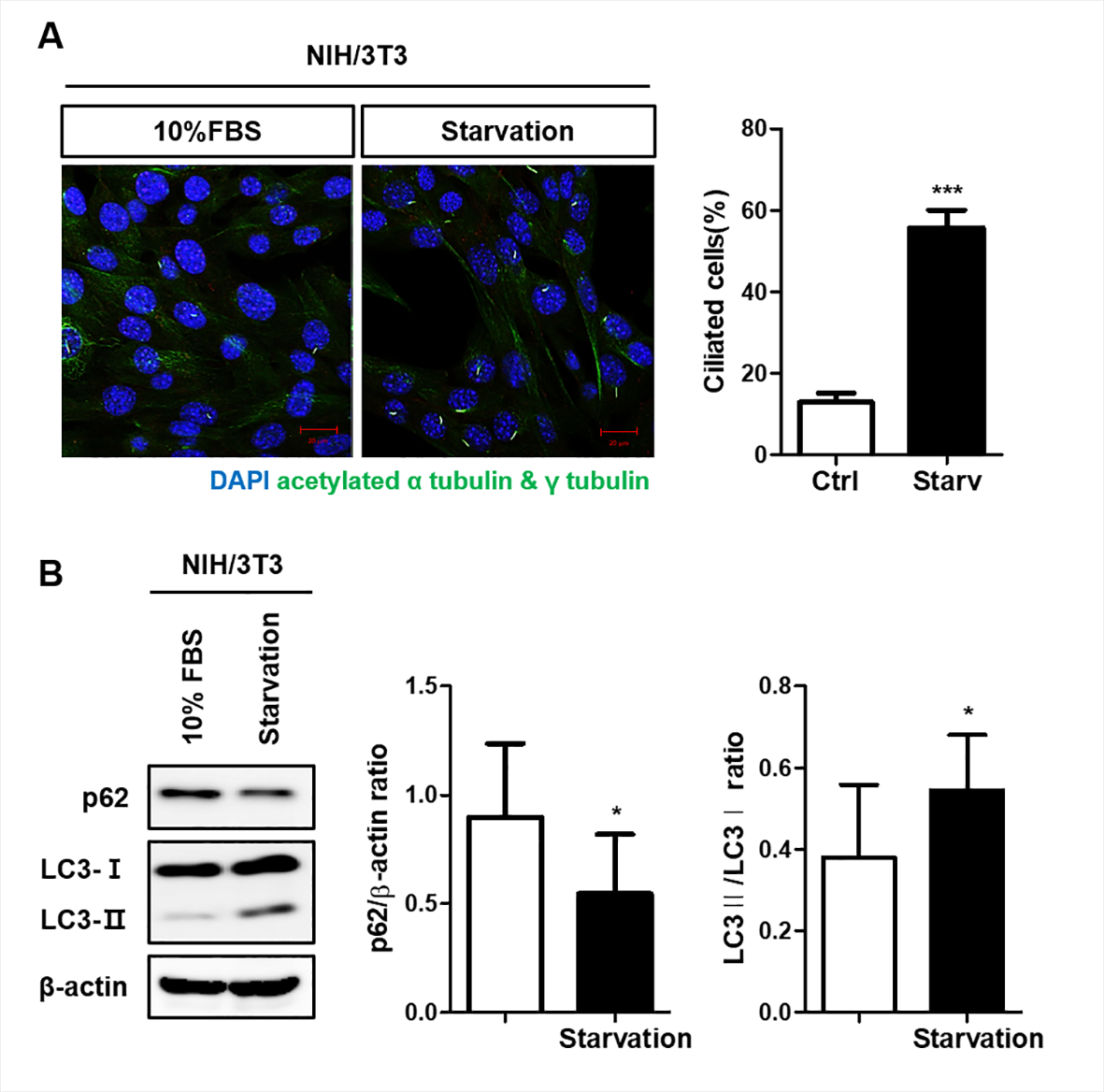

Supplement: FIGURE S1 — Simultaneous induction of autophagy and ciliogenesis under serum starvation. (A) Increased number of ciliated cells by serum starvation induced by reducing FBS to 0.5% for 24 h. The number of ciliated cells were quantified by cilia-to-nucleus ratio. (B) Changes of autophagy flux through monitoring LC3 conversion into lipidated form (LC3-II) and the level of cargo protein p62 in starved condition (0.5% FBS, 24 h). All data were obtained from a minimum of three independent experiments. Statistics analyzed by two-tailed t-tests, and P < 0.05 was considered statistically significant (∗P < 0.05, ∗∗P < 0.01, ∗∗∗P < 0.001). [file Image_1.tif]
